# Carbon ion radiotherapy for unresectable localized axial soft tissue sarcoma

**DOI:** 10.1002/cam4.1679

**Published:** 2018-07-20

**Authors:** Reiko Imai, Tadashi Kamada, Nobuhito Araki, and the Working Group for Carbon Ion Radiotherapy for Bone and Soft Tissue Sarcomas, Satoshi Abe, Yukihide Iwamoto, Toshifumi Ozaki, Hirokazu Chuman, Hiroaki Hiraga, Toru Hiruma, Noriaki Kameda, Chihiro Kanehira, Mitsunori Kaya, Rikuo Machinami, Akihiko Matsumine, Seiichi Matsumoto, Hideo Morioka, Yoshihiro Nishida, Kazuhisa Takahashi, Masazumi Tsuneyoshi, Takehiko Yamaguchi, Tsukasa Yonemoto

**Affiliations:** ^1^ Hospital of the National Institute of Radiological Sciences Quantum and Radiological Science and Technology Chiba Japan; ^2^ Department of Orthopedic Surgery Ashiya Municipal Hospital Hyougo Japan

**Keywords:** carbon ion radiotherapy, charged particle therapy, radiotherapy, soft tissue sarcoma

## Abstract

Carbon ion radiotherapy is known for its high‐precision dose distribution and high biological effectiveness. We evaluated the results of carbon ion radiotherapy in 128 patients with unresectable localized axial soft tissue sarcoma at a single institution. The patients’ median age was 54 years, and the median follow‐up period was 49.4 (range 6.4‐146.4) months. The median tumor volume was 356 cm^3^. The 5‐year local control, overall survival, and disease‐free survival rates were 65%, 46%, and 39%, respectively. In the univariate analysis, tumor volume, local control, and incidences of metastases were significantly related to overall survival. In the multivariate analysis, tumor volume and local control were significantly related to overall survival. We did not find any factors related to local control. Five patients required surgical intervention because of adverse events in the bones. Carbon ion radiotherapy may be a treatment option for unresectable axial soft tissue sarcoma.

## 1 INTRODUCTION

The main treatment option for soft tissue sarcoma (STS) is resection, and complete resection plays an important role in achieving positive oncologic results.^1^, ^2^ Radiotherapy (RT), in the case of STS, plays an auxiliary role to surgery and is applied preoperatively and/or postoperatively.^3^, ^4^ The main modalities of perioperative RT are external irradiation, intraoperative RT, and brachytherapy using photon beams. Several studies have focused on preoperative and/or postoperative irradiation for sarcomas, predominantly those of the extremities.^3^

However, few studies have focused on definitive RT without surgery for STSs.^5^, ^6^, ^7^, ^8^ As most kinds of STSs are radio‐resistant in nature, high‐dose irradiation is essential in controlling them; however, sometimes, it is difficult to achieve high‐dose irradiation due to the tumor location being close to organs sensitive to irradiation. The current form of high‐precision radiation therapy, which has been developed over several decades, can be used to provide higher irradiated doses to the target and lower irradiated doses to the normal tissues surrounding the tumor, unlike the form of radiation therapy offered before that.^9^, ^10^ As one of the more developed modalities, charged particle therapy is promising for sarcomas, as per various reports^10^, ^11^, ^12^, ^13^; currently, proton therapy and carbon ion radiotherapy (CIRT) are available worldwide. Charged particle beams have a special Bragg peak profile, allowing for more effective dose distribution compared to photon beams. Proton therapy makes the best use of this physical advantage for application in adjuvant and neoadjuvant RT in STS of the extremities, as it allows for an increase in irradiated doses and decreases in the incidences of late morbidities associated with RT, such as bone fractures.^10^ Carbon ion beams have higher biological effectiveness than proton beams do, and CIRT has been used as definitive RT for sarcomas.^11^, ^12^, ^13^ We reviewed cases with localized unresectable STSs treated with CIRT at a single institute and evaluated the effectiveness and safety of the treatment. To our knowledge, this is the first report showing oncologic results of CIRT for STSs in over 100 patients.

## 2 PATIENTS AND METHODS

Between April 2000 and March 2015, two in‐house clinical studies focusing on CIRT for unresectable bone tumors and STSs were performed at a single institution. The main eligibility criteria of these studies were as follows: (a) the tumor judged as being medically unresectable by surgeons, (b) histology confirming the presence of sarcoma, (c) absence of metal instrumentation affecting treatment planning, (d) tumor being located below C2 (sarcomas of the head and face were excluded), and (e) ECOG performance status of 0‐2. (f) Cases of radiation‐induced sarcoma except patients with recurrent tumors who had received irradiation previously, via X‐ray beams or charged particle beams. The first study was a phase I/II fixed‐dose study, and, based on the findings, the basic workable dose of CIRT for sarcomas was set at 70.4 Gy (relative biological effectiveness [RBE]). The second study was performed under similar conditions. The pathologies of all the tumors were reviewed by an in‐house pathologist. However, the pathological grade, using the French Federation Nationale des Centers de Lutte Contre le Cancer (French Federation of Comprehensive Cancer Centers, FNCLCC) system, was not defined in all the tumors, due to the lack of an adequate amount of specimens or unknown reasons. Patients with desmoid tumors or dermatofibrosarcoma protuberans were not included as candidates for CIRT. We analyzed the results of patients with unresectable axial STS treated with CIRT according to the protocol.

### 2.1 Carbon ion radiotherapy

Carbon ion radiotherapy was performed with definitive intent. Neoadjuvant or adjuvant CIRT was not planned for any tumors. Carbon ion beams were generated by a synchrotron, and accelerated energies of 290, 350, 400, and 430 MeV/n were available. For axial sarcomas, 350 and 400 MeV/n beams were generally used. As these energy beams had a water‐equivalent depth range of 15‐28 cm, the effective size of the irradiated target was determined at almost 15 cm. If the maximum length of the tumor exceeded 15 cm, a patch‐based technique was used, where the target field was further divided into two fields, and irradiation was administered to each field sequentially. The Bragg peak of the beams was modulated to fit the tumor shape using a pair of wobbler magnets, beam scatterers, ridge filters, collimators, and compensation boluses. A respiratory gating system was employed. Carbon ion doses are expressed as photon‐equivalent doses in Gy (RBE) and were defined as the physical dose multiplied by the carbon ion RBE.^14^ The biological flatness of the spread‐out Bragg peak was normalized using the surviving fraction of human salivary gland tumor cells at the distal spread‐out Bragg peak region, resulting in a carbon ion RBE of 3.

**Figure 1:**
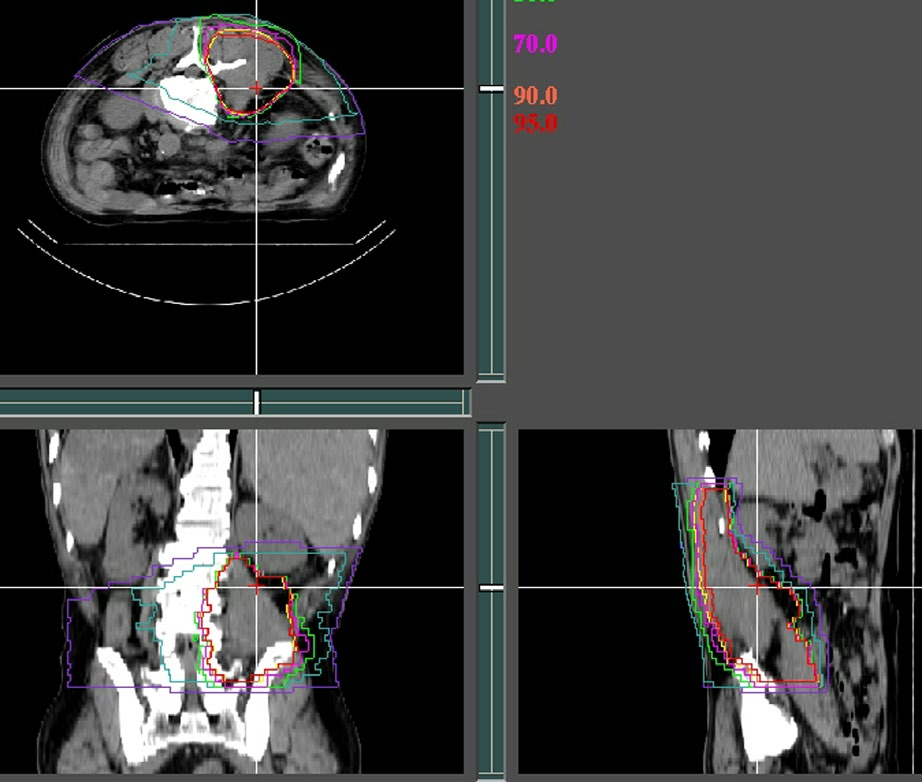
Dose distribution of carbon ion beams in a retroperitoneal grade 3 pleomorphic spindle cell sarcoma (red line, 90% isodose of the prescribed dose 70.4 Gy (RBE)/16 fractions/4 wk with three ports). The planning target volume was 500 cm^3^

To immobilize patients, low‐temperature thermoplastic shells and body rests were used. A set of 1‐5 mm thick computed tomographic (CT) images was taken for treatment planning. Magnetic resonance imaging (MRI) and CT images, using contrast medium, and, if available, methionine or fluorodeoxyglucose positron‐emission tomographic images, were used to evaluate the invasion of the tumor to the surrounding normal tissues. The clinical target volume (CTV) included the tumor's potential spreading area and the planning target volume (PTV) basically included the CTV, with an additional 1‐5 mm margin depending on the target's shape and size, and the selection of collimators. The setting technique of PTV is not always the same as the setting technique for the photon irradiation field. Irradiation was employed with a minimum of three ports (Figure 1). One port, per day, was used, and treatment was usually administered on four consecutive days a week. According to the protocols, the total setting dose was 64.0, 70.4, or 73.6 Gy (RBE) in 16 fractions for soft tissue sarcomas. Some tumors abutting the spinal cord received 64.0 Gy (RBE). Irradiation in the early stage with 73.6 Gy (RBE) in 16 fractions had reportedly resulted in the development of severe late events such as skin ulcers requiring surgery, leading to treatment cessation.^13^ Hence, a workable dose with a minimum occurrence of adverse events at 70.4 Gy (RBE) in 16 fractions was selected. All patients signed an informed consent form, and this study was approved by the local Institutional Review Board as 16‐024.

### 2.2 Follow‐up and statistical analyses

The follow‐up period was estimated from the day when the initial CIRT treatment session started. Patients were monitored through physical examinations, CT, and MRI. Initial follow‐up imaging examinations were performed after the completion of all CIRT sessions, and follow‐ups were conducted every 3‐4 months thereafter, generally using CT and MRI with a contrast medium, in an alternating fashion. In cases in which patients were unable to travel to our hospital, their latest medical reports and CT/MRI images were sent to us by their local hospitals. Local control (LC) was generally defined as no increase in tumor volume on two consecutive MRI or CT scans. Local recurrence was defined as the enlargement of the tumor inside the irradiation field or appearance of new tumors connecting to the irradiated field, suggesting the failure of the evaluation of the CTV. The toxicities attributable to RT were scored using the Common Toxicity Criteria, version 3.0 (United States National Cancer Institute, Bethesda, MD, USA).

The overall survival (OS), disease‐free survival (DFS), and LC rates were determined by the Kaplan‐Meier method, using Prism 5 (version 5.0; GraphPad Software Inc., San Diego, CA, USA). The log‐rank test was used for individual comparisons using the same software. Multivariate analysis was performed using SPSS version 23 (IBM Corp., Armonk, NY, USA). A value of *P *<* *0.05 was considered statistically significant.

## 3 RESULTS

Between June 2000 and March 2015, 128 patients with localized unresectable STSs of the axis were treated with CIRT, at a single institute. The details of the patients’ characteristics are summarized in Table 1. The pathologies of all the tumors were reviewed by the in‐house pathologist. While the FNCLCC pathological grades were not determined for all the tumors, in 68 cases, they were cleared. The study sample comprised 70 male and 58 female patients, with a median age of 54 years (range, 14‐82 years). All patients were followed up for at least 1 year or until death. The median tumor volume was 356 cm^3^ (range, 16‐1850 cm^3^) and median maximum diameter was 9 cm (range, 3‐18 cm).

**Table 1:** Patients’ characteristics

Characteristic (n = 128)	No. of patients
Median age (range), y	54 (14‐82) y
Male:female ratio	70:58
Tumor type	
Primary tumor with no prior surgery	74
Recurrent tumor after resection	32
Residual tumor after resection	4
Metastatic tumor	18
Irradiation site	
Subdeep (back, neck, gluteus muscle)	45
Deep site (retroperitoneum, pelvis, chest wall, abdominal wall)	123
Median size (range), cm^3^	356 (16‐1850) cm^3^
~200	37
200‐500	47
500‐1000	34
1000~	10
Histology	
UPS	29
MPNST	15
Liposarcoma^b^	12
Synovial sarcoma	14
Leiomyosarcoma	10
Others	48
Grade^a^	
G1	14
G2	6
G3	48
Unknown	60
Total irradiation dose (Gy [RBE] in 16 fractions)	
64.0	8
70.4	115
73.6	5
Chemotherapy before CIRT	
Yes	50
No	78

MPNST, malignant peripheral nerve sheath tumor; RBE, relative biological effectiveness; UPS, undifferentiated pleomorphic sarcoma.

a The French Federation of Comprehensive Cancer Centers (FNCLCC) system.

b Well differentiated in three patients, others in nine patients.

### 3.1 Tumor control

**Figure 2:**
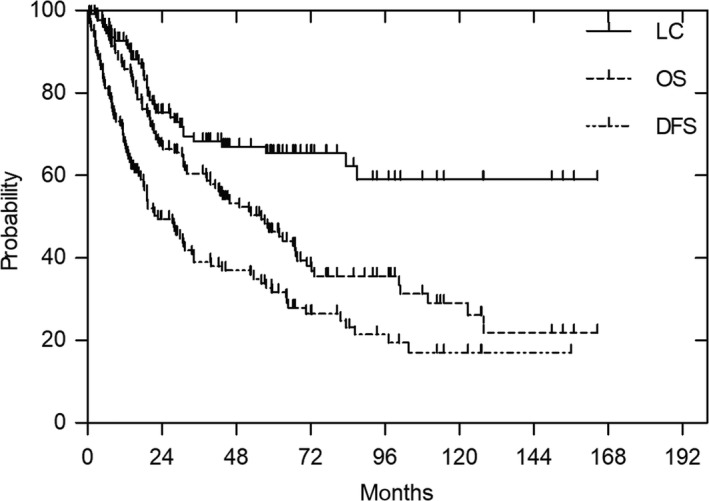
Local control, overall survival, and disease‐free survival rate in 128 patients

Of the 128 tumors, the 3‐year and 5‐year LC rates were 68% and 65%, respectively (Figure 2). Local recurrence was observed in 38 cases, with 26% of local recurrences happening in 1 year after CIRT, 74% in 2 years and 89% in 3 years. The two local recurrence cases at 87 and 83 months after CIRT were both malignant peripheral nerve sheath tumors (MPNST). In the recurrent cases, in‐field recurrence was observed in 25 patients, and 20% and 16% of in‐field recurrences involved MPNST and synovial sarcoma, respectively. The tumor size (lower or higher than 500 cm^3^), total irradiated dose (lower or higher than 70.4 Gy [RBE]), disease status (primary or nonprimary disease), presence of metastases (yes or no), sex, and age (younger or older than 55 years) were not significant in the univariate analysis. We did not find any significant factors affecting the LC rate. Of the 68 patients with a clarified histological grading, LC was not related to tumor grade. According to histology, 5‐year LC rates were 66, 52, 42, and 90% in undifferentiated pleomorphic sarcoma, MPNST, synovial sarcoma, and liposarcoma, respectively.

### 3.2 Survival

The median survival was 42 months for the 128 patients, with a range of 6‐146 months. Seventy‐eight patients died during the evaluation period, and 43 patients survived for over 5 years. The median survival of the patients who died was 22 months, ranging from 2.3 to 128 months. The 3‐year and 5‐year OS rates were 60% and 46%, respectively (Figure 2). In the univariate analysis, local control (*P* = 0.0218), tumor size (lower or higher more than 500 cm^3^) (*P* = 0.011), and the presence of metastasis (*P* = 0.0355) showed a significant association with OS rate. The prognostic factors analyzed in the univariate analysis are summarized in Table 2. In the multivariate analysis, local control (*P* = 0.012), and tumor size (*P* = 0.02) were significantly associated with OS. OS was worse in patients receiving chemotherapy prior to CIRT (*P* = 0.03). In terms of the FNCLCC grades, where available, there were significant differences in the OS between patients with grade 1 and grade 2 (*P* = 0.049), and those with grade 1 and grade 3 (*P* = 0.0016), based on univariate analysis (Table 3).

**Table 2:** Univariate analysis of the 5‐y overall survival

	# Patients	5‐y OS, %	*P*‐value
Total # patients	128	46	
Age (y)			
<55	60	48	0.46
≥55	68	43	
Sex			
Male	70	53	0.234
Female	58	39	
Local recurrence			
Yes	37	29	0.0218
No	91	54	
Metastases			
Yes	71	37	0.0355
No	57	59	
Tumor presentation			
Primary	74	52	0.710
Others	54	40	
Target volume (cm^3^)			
<500	84	51	0.0114
≥500	44	38	
Total dose (Gy RBE)			
<70.4	8	50	0.45
≥70.4	120	46	
Chemotherapy before CIRT			
Yes	50	40	0.03
No	78	51	

#, number; CIRT, carbon ion radiotherapy; Gy RBE, Gray Relative Biological Effectiveness; LC, local control; OS, overall survival.

**Table 3:** Univariate analysis of the 5‐y overall survival according to the FNCLCC grades in patients for whom these data were available

	# Patients	5‐y OS, %	*P*‐value
	60	51.2	
FNCLCC Grade			
G1	14	85	0.0498
G2	6	67	
FNCLCC Grade			
G1	14	85	0.0016
G3	48	39	

FNCLCC, French Federation Nationale des Centers de Lutte Contre le Cancer (French Federation of Comprehensive Cancer Centers, FNCLCC); OS, overall survival.

**Figure 3:**
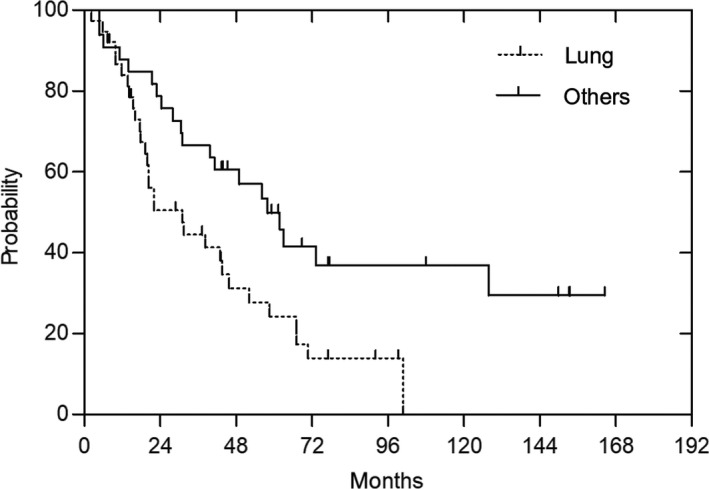
Overall survival in patients with metastases according to the first metastatic site. In patients in whom the first metastasis sites were the lungs, the OS was significantly lower than those in whom the first metastasis sites were not the lungs (*P* = 0.01)

The DFS rates at 3 years and 5 years were 39% and 32%, respectively (Figure 2). Distant metastases were observed in 71 patients, and the first metastasis site was the lung in 38 patients. The median time to the occurrence of metastasis was 12 months, with a range of 0.7‐103 months. In patients in whom the first metastasis sites were the lungs, the OS was significantly lower than those in whom the first metastasis sites were not the lungs (*P* = 0.01) (Figure 3).

### 3.3 Adverse events

Late adverse events—grade 3 and higher—were observed in four patients. A grade 3 spinal cord injury was observed in one patient, grade 3 peripheral nerve injury in one, grade 4 colon injury requiring colostomy in one, and grade 3 skin injury also in one patient. The patient with a grade 3 spinal cord injury received CIRT twice due to local recurrence. In two patients, Brown‐Séquard syndrome (grade 2) was observed with sensory disturbances but no motor nerve dysfunction.

## 4 DISCUSSION

Complete resection in cases of STS is important in achieving good oncologic results,^1^, ^2^, ^15^ but only 40%‐60% of deep‐seated STS can be completely resected.^15^, ^16^, ^17^, ^18^, ^19^ Several studies have reported that the presence of positive margins can lead to worse LC and OS than those associated with negative margins.^1^, ^2^, ^15^, ^16^, ^17^, ^18^ Since resection is essential treatment for STS, a small number of reports have focused on unresectable cases.^5^, ^6^, ^7^, ^8^ Smith et al reported that, in 19 unresectable pediatric and young adult nonrhabdomyosarcoma STS patients, local progression was observed in 13 patients after definitive RT, at a median dose of 55.2 Gy. The median time to local progression was 1 year, and actual LC and OS rates at 5 years were 40% and 37%, respectively.^5^ LeVay et al^20^ reported that of the 321 patients receiving curative‐intent treatment for STS of the extremities, torso, head, and neck, excluding that of the retroperitoneum, 17 patients refused definitive surgery; they were treated with definitive RT, and their 5‐year cause‐specific survival was 35%, with a local relapse rate of 77% even though they were resectable cases. Slater et al^7^ reported that, in 72 unresectable STS cases treated with photons alone, or photons and neutrons for at least part of the treatment, the 5‐year LC rate was 29%, with 48% of in‐field recurrences occurring within 2 years. In a study by Kepka et al, 112 STS patients, with tumors located in the extremities in approximately half of them, underwent RT for gross disease, after surgical biopsy or radical surgery. The 5‐year LC, OS, and DFS rates were reportedly 45%, 35%, and 24%, respectively.^6^ In our series, the 5‐year LC, OS, and DFS rates were 65%, 46%, and 32%, respectively. Regarding LC rates, our data were superior to previous data. Kepka et al^6^ mentioned that LC rates at 5 years were 51, 45, and 9% for tumors <5 cm, 5‐10 cm, and >10 cm, respectively. In our series, LC rates at 5 years for tumors less than 5 cm (n = 25), 5‐10 cm (n = 60), and >10 cm (n = 43) were 61%, 67%, and 65%, respectively. The tumor size did not influence LC rates in our series. It could be explained that better dose distribution of carbon ion beams led to a higher dose irradiation to tumors. A biologically equivalent dose of 10 (BED10) of 70.4 Gy (RBE) in 16 fractions using carbon ion beams was 101 Gy (RBE)10 and higher than those in previous studies using photon beams. Kepka et al^6^ showed a relation between local control and tumor size using lower (<63 Gy) and higher (≥63 Gy) doses. LC rates at 5 years for tumors less than 5 cm, 5‐10 cm, and greater than 10 cm at the total doses of < 63 Gy and ≥63 Gy were 22% and 72%, 49% and 42%, and 0% and 25%, respectively, revealing that large tumors will need irradiation doses higher than 63 Gy, despite the proximity of sensitive organs in larger tumors. Twenty‐six percent of patients who received doses of 68 Gy or more had major complications, whereas only 8% of those who received doses less than 68 Gy had major complications.^6^ Nonetheless, higher irradiation doses are accompanied by a higher local control rate. In our cases, even if the tumor diameter was greater than 10 cm, tumors could receive higher dose irradiation compared to that of previous radiation treatment using sharpness of the carbon ion beams to avoid irradiation to critical organs. In addition, although no prognostic factors for local control were found in this study, the majority of our cases showed histological grade 3 and may have died before the incidence of local recurrence. To improve understanding regarding the relationship with tumor grade, a competing risk factor analysis will be useful but will require a sufficient number of patients with various grade tumors.

Carbon ion radiotherapy could be more effective in larger STS. Using carbon ion beams will achieve higher irradiation doses with less complications due to its special physical profile. In our report, 38 cases had local recurrence, and 89% of local recurrences occurred in 3 years, similar to that of previous reports.^6^, ^7^ In recurrent cases, in‐field recurrence was observed in 25 patients, and 20 and 16% of in‐field recurrences were MPNST and synovial sarcoma, respectively. The total dose of CIRT was determined by phase I and phase I/II clinical trials. However, the findings in the current study indicate the possibility of dose escalation in selected cases like MPNST and synovial sarcoma.

Interestingly, at 35%‐40%, the 5‐year OS and DFS were not significantly different in the above‐mentioned cases of unresectable tumors and our cases.^5^, ^6^, ^7^, ^20^ O'Donnell et al^21^ stated that in surgical cases, tumors with positive margins were almost certainly more biologically aggressive than those with negative margins and were associated with a greater risk of both local and systemic recurrence. If biologically aggressive STSs can be classified in this manner, minimally invasive treatments such as CIRT may be an option for their treatment. In our series, OS was lower in patients receiving chemotherapy before CIRT than those not receiving it. This study is a retrospective study and it is difficult to analyze the reason the patients not receiving chemotherapy before CIRT had better OS rate; however, the maximum tumor diameter (8 cm vs 11.5 cm) was smaller and the number of grade 1 tumors (13 cases vs 1 case) was greater in the latter.

Progress in the technology associated with various perioperative RT modalities has strengthened their potential in the surgical treatment of axial STS, especially along with margin setting.^22^, ^23^ However, for cases of unresectable axial STSs, there are still not enough data concerning the efficacy of each of the modalities. At this stage, CIRT could be a treatment option for unresectable localized axial STSs.

## CONFLICT OF INTEREST

None declared.

## APPENDIX 1

### 1.1

*Working Group for Carbon Ion Radiotherapy for Bone and Soft Tissue Sarcomas:* Satoshi Abe: Department of Orthopaedic Surgery, Teikyo University School of Medicine, Tokyo, Japan; Yukihide Iwamoto: Kyusyu Rosai Hospital, Japan Organization of Occupational Health and Safety, Fukuoka, Japan; Toshifumi Ozaki: Department of Orthopaedic Surgery, Okayama University Graduate School of Medicine, Okayama, Japan; Hirokazu Chuman: Department of Musculoskeletal Oncology and Rehabilitation, National Cancer Center, Tokyo, Japan; Hiroaki Hiraga: Department of Musculoskeletal Oncology, Hokkaido Cancer Center, Hokkaido, Japan; Toru Hiruma: Department of Orthopaedic Surgery, Kanagawa Cancer Center, Kanagawa, Japan; Noriaki Kameda: Chiba Cytology Laboratory center, Chiba, Japan; Chihiro Kanehira: Department of Radiology, The Jikei University School of Medicine, Tokyo, Japan; Mitsunori Kaya: Department of Orthopaedic Surgery, Sapporo Medical University of Medicine, Hokkaido, Japan; Rikuo Machinami: Department of Diagnostic Pathology, Kawakita General Hospital, Tokyo, Japan; Akihiko Matsumine: Department of Orthopaedic Surgery, University of Fukui Faculty of Medical Sciences, Fukui, Japan; Seiichi Matsumoto: Department of Orthopaedic Surgery, Japanese Foundation for Cancer Research, Tokyo, Japan; Hideo Morioka: Department of Orthopaedic Surgery, Keio University School of Medicine, Tokyo, Japan; Yoshihiro Nishida: Department of Orthopaedics/Rheumatology, Nagoya University Graduate School of Medicine, Aichi, Japan; Kazuhisa Takahashi: Department of Orthopaedic Surgery, Graduate School of Medicine, Chiba University, Chiba, Japan; Masazumi Tsuneyoshi: Department of Diagnostic Pathology, Fukuoka Sanno Hospital, Tokyo, Japan; Takehiko Yamaguchi: Department of Diagnostic Pathology, Dokkyo Medical University, Tochigi, Japan; Tsukasa Yonemoto: Department of Orthopaedic Surgery, Chiba Cancer Center, Chiba, Japan.
